# MMP13 mediates cell cycle progression in melanocytes and melanoma cells: in vitro studies of migration and proliferation

**DOI:** 10.1186/1476-4598-9-201

**Published:** 2010-07-28

**Authors:** Svenja Meierjohann, Anita Hufnagel, Elisabeth Wende, Markus A Kleinschmidt, Katarina Wolf, Peter Friedl, Stefan Gaubatz, Manfred Schartl

**Affiliations:** 1 Department of Physiological Chemistry I, Biocenter, University of Wurzburg, Am Hubland, 97074 Wuerzburg, Germany; 2 Institute for Medical Microbiology, Medical University of Hannover, Hannover, Germany; 3 Physiological Chemistry, University Medical Center Utrecht, University of Utrecht, Netherlands; 4 Department of Cell Biology (283) NCMLS, Radboud University Nijmegen Medical Centre, P.O. Box 9101, 6500 HB Nijmegen, The Netherlands

## Abstract

**Background:**

Melanoma cells are usually characterized by a strong proliferative potential and efficient invasive migration. Among the multiple molecular changes that are recorded during progression of this disease, aberrant activation of receptor tyrosine kinases (RTK) is often observed. Activation of matrix metalloproteases goes along with RTK activation and usually enhances RTK-driven migration. The purpose of this study was to examine RTK-driven three-dimensional migration of melanocytes and the pro-tumorigenic role of matrix metalloproteases for melanocytes and melanoma cells.

**Results:**

Using experimental melanocyte dedifferentiation as a model for early melanomagenesis we show that an activated EGF receptor variant potentiates migration through three-dimensional fibrillar collagen. EGFR stimulation also resulted in a strong induction of matrix metalloproteases in a MAPK-dependent manner. However, neither MAPK nor MMP activity were required for migration, as the cells migrated in an entirely amoeboid mode. Instead, MMPs fulfilled a function in cell cycle regulation, as their inhibition resulted in strong growth inhibition of melanocytes. The same effect was observed in the human melanoma cell line A375 after stimulation with FCS. Using sh- and siRNA techniques, we could show that MMP13 is the protease responsible for this effect. Along with decreased proliferation, knockdown of MMP13 strongly enhanced pigmentation of melanocytes.

**Conclusions:**

Our data show for the first time that growth stimuli are mediated via MMP13 in melanocytes and melanoma, suggesting an autocrine MMP13-driven loop. Given that MMP13-specific inhibitors are already developed, these results support the evaluation of these inhibitors in the treatment of melanoma.

## Background

Growth factors control the fate of many cell types in the body and usually stimulate proliferation, survival and motility in cells that express the adequate receptor on their surface. Therefore, availability of growth factors and growth factor receptors must be tightly regulated on multiple levels to prevent aberrant growth. However, many tumors have developed mechanisms that render them independent of exogenous growth factors. One mechanism is the development of autocrine loops. Multiple tumors including melanoma produce high amounts of EGF, TGF-α, PDGF, or bFGF which accelerates tumor growth and goes along with a reduced patient survival [1-5]. Furthermore, mutations in growth factor receptors can generate continuous growth signals, e.g. in glioblastoma, breast, ovarian, prostate and lung squamous cell carcinomas, where the truncated epidermal growth factor receptor version vIII is expressed [6-8]. The oncogenic EGFR variant *Xiphophorus *melanoma receptor kinase (Xmrk) is also permanently active due to mutations that result in constitutive dimerization of this receptor tyrosine kinase [9,10]. Xmrk is the cause for highly aggressive melanoma in the *Xiphophorus *fish tumor model. It constitutes a very efficient oncogene that induces the steps necessary for melanoma formation *in vivo *in the fish model [11-13] and also *in vitro *in mammalian melanocytes [14,15].

Of the different steps required for tumor formation and progression, induction of cell motility and survival in the extracellular matrix are considered to be crucial prerequisites for a tumor cell to become metastatic. When a melanocyte succeeds to leave its natural epidermal environment and invades the dermis it has to face a new surrounding, consisting mainly of collagen. The lack of a proper cell-matrix attachment leads to an anoikis-like state and drives these cells into apoptosis [16,17]. Activation of growth factor receptors, however, can both protect the cells from apoptosis and induce migration in a three-dimensional collagen environment [17-19]. Most migrating cells express either membrane-bound or secreted matrix metalloproteases (MMPs) at the cell front that digest the matrix and open space for the forward pushing cell body. MMPs are commonly upregulated after growth factor stimulation [20]. Although the best studied targets of these proteases are various matrix components, a growing body of evidence reveals the importance of MMP-dependent cleavage of other extra- and intracellular substrates that have various cellular effects [21-23].

Here, we take advantage of the well defined transforming abilities of the oncogene *xmrk *and use it as model to analyze the cancer-inducing functions of receptor tyrosine kinases (RTK). In order to concentrate on RTK-driven effects alone without influences from secondary tumor-derived effects we are using Xmrk-expressing melanocytes (melan a) rather than melanoma cells. Activation of Xmrk leads to transformation of these cells and induces key features of the neoplastic phenotype of melanoma cells [14]. One of these key features is the occurrence of dedifferentiation, which can be directly visualized by decresed pigmentation and reduced tyrosine levels after Xmrk activation [15]. Besides dedifferentiation and unlimited proliferation, Xmrk has been previously reported to induce cellular migration of melanocytes in a two-dimensional migration assay [24] and mediate cell survival in three-dimensional collagen lattices [17]. In this study, we investigated the three-dimensional migration behaviour. We found that Xmrk activation induced melanocyte migration in an amoeboid manner which is entirely independent of MMP activity. Instead, blocking MMPs with a broadband inhibitor mix stalled cell proliferation. The protease responsible for the proliferation effect was MMP13, as demonstrated by RNA knockdown experiments. Importantly, MMP13 was also found to be necessary for the proliferation of the human melanoma cell line A375.

## Results

### EGF stimulation of melanocytes leads to MAPK- and PI3K-independent migration on collagen

**Figure 1:**
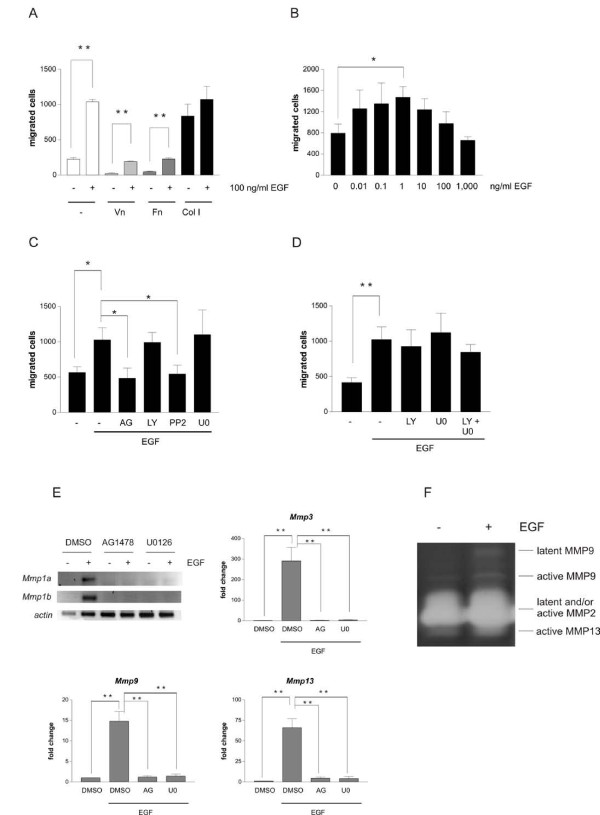
**EGF induces melanocyte motility on collagen I and the expression of several matrix metalloproteases**. A-D: Hm cells were serum-starved for 24 h and seeded onto the upper chamber of transwell migration inlays. The transwell migration assays were stopped after 12 h. Cells were stained with crystal violet and the number of transmigrated cells was counted. A: Hm cells were seeded onto uncoated transwell migration inlays or inlays previously coated with vitronectin (Vn), fibronectin (Fn) or collagen I (Col I). Where indicated, EGF were applied to the lower chamber. B: Transwell migration assay of Hm cells seeded onto collagen I-coated inlays. The indicated concentrations of EGF were applied to the lower chamber. C-D: Transwell migration assay of Hm cells seeded onto collagen I-coated inlays and stimulated with 1 ng/ml EGF, applied to the lower chamber. Cells were additionally treated with AG1478 (AG), LY294002 (LY), PP2, U0126 (U0) (C) or a combination of LY294002 and U0126 (LY + U0) (D). **: p < 0.001 (Student's t test, paired, two-tailed). E: Hm cells were serum-starved for 24 h and subsequently left untreated or treated with 100 ng/ml EGF in presence or absence of AG1478 or U0126. After 8 h, cells were harvested and reverse transcription-PCR (in case of *Mmp1a *and *Mmp1b*) or realtime-PCR analysis (in case of *Mmp3*, -9 and -13) were performed. Realtime-PCR data were normalized to expression of actin. **: p < 0.001 (Student's t test, paired, two-tailed). F: Zymographic analysis of the supernatant of starved or EGF-treated Hm cells after two days of stimulation.

To monitor the effects of signalling of the oncogenic RTK Xmrk we used HERmrk-transgenic melanocytes ("melan-a Hm") that transgenically express a chimeric protein consisting of an extracellular EGFR- and an intracellular Xmrk domain. It is important to note that these cells do not express endogenous EGFR [15]. The chimeric receptor displays the same intracellular signalling as Xmrk and in addition allows EGF induction instead of permanent activation. To find out which matrix components are suitable for migration of melan-a Hm we first performed a modified Boyden chamber assay on transwell inlays that were either left uncoated or were precoated with vitronectin, fibronectin, or collagen I. We used 100 ng/ml of EGF, which is the concentration that proved to be optimal for migration on uncoated transwell inlays [24]. The results demonstrate that only uncoated and collagen-coated membranes constitute a good migration substrate for the cells (Figure 1A). However, significant EGF-induced migration on collagen I was only noted with reduced amounts of EGF as stimulus (1 ng/ml, Figure 1B).

For evaluating which downstream components are important for collagen-mediated cell migration, we performed migration experiments at 1 ng/ml EGF in the absence or presence of the following small molecule inhibitors: AG1478 (blocks kinase activity of EGFR and orthologues including Xmrk and HERmrk), LY294002 (blocks PI3K), PP2 (blocks SRC family kinases) and U0126 (blocks MEK). Inhibition of SRC kinases and HERmrk itself led to a reduction in cell motility (Figure 1C), which is in accordance with previous observations monitoring two-dimensional migration in absence of collagen [24]. Single and combined inhibition of PI3K and MAPK pathways, however, revealed that both pathways are dispensable for 2D migration of HERmrk melanocytes (Figure 1C). However, both inhibitors efficiently blocked the respective pathways at the applied concentration of 10 μM (Additional file 1, Figure S1A) The same observation was made when an independent MAPK inhibitor, namely PD184352, was combined with the PI3K inhibitor (Additional file 1, Figure S1B and, as inhibitor control: Additional file 2, Figure S2A, right lanes).

### EGF stimulation induces several MMPs in a MAKP-dependent manner

As interaction of cells with matrix components often induces both the secretion of matrix proteases and the secretion of extracellular matrix components, we screened for the expression of both groups of genes in response to EGF. EGF strongly upregulated the transcripts of matrix metalloproteases *MMP1a*, -*1b*, -*3*, -*9 *and -*13*, which are not or only slightly expressed in absence of EGF. The other investigated proteases or the matrix components collagen I, IV, laminin and fibronectin were not induced (Table 1). Inhibiting either HERmrk or MEK with two independent inhibitors for each pathway totally abolished the induction of each of the MMPs (Figures 1E and Additional file 2, Figure S2A-B). The fact that MMP induction depends on the MAPK pathway, which itself is not involved in the EGF induced migration (see above), indicates that MMPs are not required for EGF-induced migration on two-dimensional collagen substrate.

**Table 1:** RNA expression of proteases and matrix components in response to longterm HERmrk stimulation.

gene	ENSEMBL reference	induction by HERmrk
*Mmp1a*	ENSMUSG00000043089	+++
*Mmp1b*	ENSMUSG00000041620	+++
*Mmp3*	ENSMUSG00000043613	+++
*Mmp7*	ENSMUSG00000018623	-
*Mmp8*	ENSMUSG00000005800	-
*Mmp9*	ENSMUSG00000017737	+
*Mmp11*	ENSMUSG00000000901	-
*Mmp13*	ENSMUSG00000050578	++
*Mmp14*	ENSMUSG00000000957	-
*Mmp15*	ENSMUSG00000031790	-
*Mmp16*	ENSMUSG00000028226	-
*Mmp24*	ENSMUSG00000027612	-
*Upa*	ENSMUSG00000021822	-
*CatD*	ENSMUSG00000007891	-
*CatL*	ENSMUSG00000021477	-
		
*Col1*	ENSMUSG00000001506	-
*Col4*	ENSMUSG00000031502	-
*Lam*	ENSMUSG00000032796	-
*Fn1*	ENSMUSG00000026193	-

The highest induction of *Mmp1a*, -1*b*, -3, -9 and -13 was seen at 8 h after EGF induction. The symbols given in the table refer to gene induction at this timepoint. +: induction 2-20-fold; ++: induction 20-100-fold; +++: induction >100-fold.

*Upa*: urokinase plasminogen activator; *CatD*: cathepsin D; *CatL*: cathepsin L; *Col1*: collagen I; *Col4*: collagen IV, *Lam*: laminin; *Fn1*: fibronectin I.

To confirm MMP activity, we performed a gelatin zymography, which is suitable for detecting active MMP2, MMP9 and MMP13. After EGF stimulation, the bands characteristic for active MMP9 and MMP13 were enhanced (Figure 1F). To find out whether induction of these MMPs is a shared feature of HERmrk and human EGFR, we also treated melan-a cells transgenic for human EGFR (melan-a HER) with EGF in absence or presence of EGFR- and MEK inhibitors and monitored transcriptional induction of MMPs. We observed enhanced expression of *Mmp1a*, -*1b*, -*3*, and -*13*, but no induction of *Mmp9*. Again, EGFR- and MEK inhibition were effective (Additional file 2, Figure S2C) and prevented the expression of all *Mmp*'s (Additional file 2, Figure S2D). The parental cell line melan-a did not respond to EGF stimulation (Additional file 2, Figure S2E).

### EGF-stimulated melanocytes migrate in an amoeboid, MMP- and MAPK-independent manner in three-dimensional collagen gels

**Figure 2:**
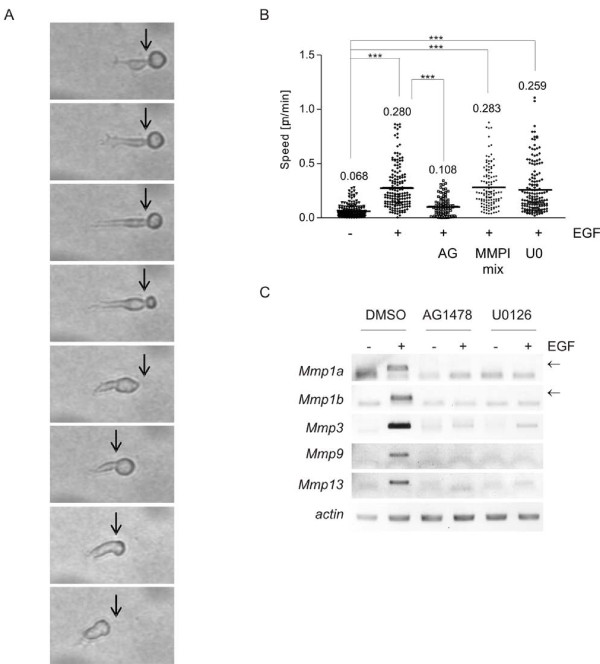
**EGF induces MMP-independent amoeboid migration in Hm cells**. A: Hm cells were embedded in a three-dimensional collagen matrix and overlaid with starving medium containing EGF. The pictures are a detail magnification of Additional file 3, Movie S1 and show the migrative behaviour of a single cell that was photographed eight times consecutively with a time interval of 4 min between the pictures. The arrow indicates areas of dense matrix where the cell has to contract its cell body. B: Analysis of the speed of Hm cells migrating in a three-dimensional collagen gel. Cells were embedded into collagen in the presence of DMSO, AG1478, U0126 and a mixture of MMPI 9/13 and GM6001 (Ilomastat). Where indicated, cells were overlaid with EGF-containing medium. Graphs display the speed of single cells that was calculated from the time-lapse movies, and the mean speed for each condition is indicated. The following numbers of cells were examined: ctrl., EGF, EGF + U0126: n = 161 (for each); EGF + AG1478: n = 78; EGF + MMP inhibitors: n = 120. ***: p < 0.0001. C: Hm cells were embedded in collagen matrix and stimulated with EGF as described above. The supernatant contained DMSO, AG1478 or U0126, respectively. After 8 h, where maximal stimulation was seen before, whole collagen gels were used for RNA isolation and RT-PCR analysis of the indicated genes. In case of *Mmp1a *and -1*b *a lower, unspecific DNA band was seen, which was presumably due to oligonucleotide multimers that can form in the absence of specific template. The specific bands for *MMP1a *and -*1b *are indicated by an arrow.

To monitor if MMP-independent migration only occurs if the melanocytes are migrating on a flat surface or whether it also takes place in three-dimensionally migrating cells, the melanocytes were analyzed by time-lapse videomicroscopy in a 3D model. The migrative behaviour of melanocytes can be best observed when cells are kept under experimental conditions that reflect the composition of the dermis. Therefore Hm cells were embedded in a three-dimensional chamber filled with fibrillar collagen and overlayed with EGF-containing medium. Cells were then monitored for 48 h. Monitoring at high resolution revealed that migrating cells squeezed through the matrix and changed their shape to a rounded or ellipsoid appearance, seemingly without degrading the matrix. This is reminiscent of amoeboid migration in melanoma and other tumor cells in three-dimensional migration model systems (Figure 2A, arrows and Additional file 3, Movie S1) [25,26].

The concept of EGF-induced amoeboid migration in melanocytes was directly addressed using broad-spectrum MMP inhibition (Ilomastat and MMP9/13 inhibitor I). Inhibition of MMPs had no effect on migration speed (Figure 2B), in agreement with the amoeboid migration which is a migration mode independent of matrix destruction by metalloproteases.

We also tested the effect of inhibiting the receptor itself and its downstream target responsible for *Mmp *upregulation, the ERK1/2 pathway. HERmrk signalling was abrogated using the EGFR inhibitor AG1478, while ERK1/2 inhibition was accomplished using the MEK inhibitor U0126. We first controlled the efficiency of both inhibitors in collagen gels. RT-PCR of all regulated *Mmp *genes demonstrated a successful inhibition of target gene induction by AG1478 and U0126 (Figure 2C). As expected, inhibition of HERmrk resulted in strongly reduced cell migration (Figure 2B). However, activation of ERK1/2 seemed to be dispensable for migration, as U0126 had no effect on cell speed (Figure 2B). This was unexpected, as ERK1 and ERK2 do not only induce *Mmp*'s, but reportedly play a role in cytoskeleton rearrangement, which is a prerequisite for motility of many cell types [27].

### MMP inhibition results in a proliferation block of EGF-treated melanocytes

**Figure 3:**
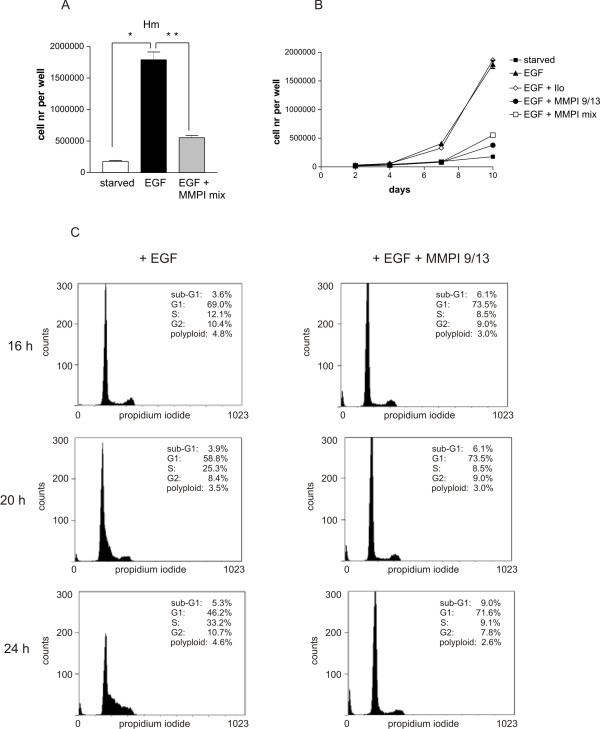
**MMP inhibition results in strongly reduced proliferation of melan-a Hm cells**. A: Hm cells were starved for two days in presence of 1.5% dialyzed FCS and were then stimulated by the addition of 100 ng/ml EGF, either in absence or presence of the MMP inhibitor mix containing MMP inhibitors MMPI 9/13 and Ilomastat. The number of cells per well after ten days of treatment is indicated. *: p < 0.05; **: p < 0.0001. B: Starved Hm cells were stimulated with EGF in absence or presence of Ilomastat, MMP9/13 inhibitor or a combination of both. Cell proliferation was measured after 2, 4, 6, 8 and 10 days. C: Cells were starved as above and were incubated with EGF in absence or presence of the MMP inhibitor 9/13. Their cell cycle state was monitored after 16, 20 and 24 h. The first accumulation of cells into S-phase is seen after 20 h. The assay was performed three times, and a representative example is shown.

Besides their contribution to ECM remodelling and invasive migration, other functions of MMPs include the proteolytic release of matrix-bound growth factors or of transmembrane proteins. This would result in auto- or paracrine outside-in-signalling. Thus, we monitored apoptosis and cell cycle progression of EGF-stimulated HERmrk transgenic melanocytes in the absence or the presence of MMP inhibitors. To examine a possible effect on cell proliferation, we stimulated starved cells with EGF in absence or presence of the MMP inhibitor mix (containing MMP inhibitors Ilomastat and MMP-inhibitor 9/13) and followed their proliferation for ten days. The inhibitors reduced cell proliferation to one third of the control (Figure 3A). When we compared the effect of single MMP inhibitors with the MMP inhibitor mix, only MMP inhibitor 9/13 proved to be effective in blocking proliferation (Figure 3B). Flow cytometry analyses demonstrated that while EGF treatment of starved HERmrk melanocytes resulted in an increase of cells in S phase after 20-24 h, no cell cycle progression was seen in presence of the MMP inhibitor 9/13 (Figure 3C). In addition, a slight increase of sub G1 cells seemed to occur in MMP inhibitor 9/13 treated cell populations, but this was not significant. Western blot analysis of cleaved caspase 3, the effector caspase downstream of intrinsic and extrinsic apoptosis stimuli, showed no apoptosis induction (Additional file 4, Figure S3A). Thus, the prevailing effect of blocking MMP9/MMP13 was the inhibition of cell cycle progression.

### Cell cycle progression of the human melanoma cell line A375 is also blocked by MMP inhibition

**Figure 4:**
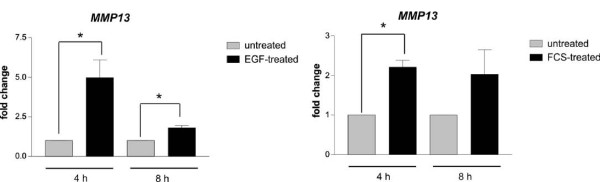
**Upregulation of human *MMP13 *in response to FCS**. A375 cells were starved for two days in presence of 0.5% dialyzed FCS and stimulated by the addition of 100 ng/ml EGF or 10% FCS. After 4 and 8 h, cells were harvested and monitored for the expression of *MMP13 *by realtime PCR. Human ribosomal S14 was used as reference gene. *: p < 0.05 (Student's t test, paired, two-tailed).

To address whether MMP-dependent cell cycle progression is also a feature of human melanoma cells, we tested the melanoma cell line A375. In contrast to starved melan-a Hm cells, starved A375 cells already expressed low amounts of *MMP1*, -*3*, -*9*, and -*13 *(data not shown). However, as we were interested in MMPs that are induced in response to growth stimulatory signals, we also analyzed the expression of these four genes in response to EGF and FCS. Under these conditions, an induction was only measured for *MMP13 *(Figure 4). Although EGFR stimulation of A375 results in pro-tumorigenic cellular effects, such as enhanced survival [28], it is not sufficient to drive the cells into cell cycle (own observations). Thus, we performed the proliferation experiments using 10% FCS as stimulant.

**Figure 5:**
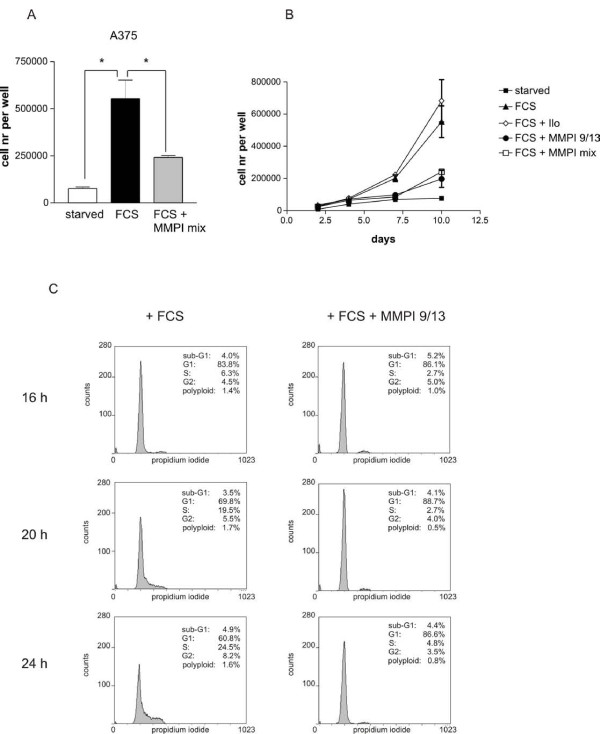
**MMP inhibition results in strongly reduced proliferation of A375 cells**. A: A375 cells were starved for two days in presence of 1.5% dialyzed FCS and stimulated by the addition of 10% FCS, either in absence or presence of the MMP inhibitor mix containing MMP inhibitors MMPI 9/13 and Ilomastat. The number of cells per well after ten days of treatment is indicated. B: Starved A375 cells were stimulated with FCS in absence or presence of Ilomastat, MMP9/13 inhibitor or a combination of both. Cell proliferation was measured after 2, 4, 6, 8 and 10 days. C: Cells were starved as above and were incubated with FCS in absence or presence of the MMP inhibitor 9/13. Their cell cycle state was monitored after 16, 20 and 24 h. The first accumulation of cells into S-phase is seen after 20 h. The assay was performed three times, and a representative example is shown.

The results mirrored the situation previously observed in melan-a Hm cells. Proliferation was blocked by the MMP inhibitor mix, and the only inhibitor responsible for this effect was MMP 9/13 (Figures 5A and 5B). The progression of starved A375 cells into S phase, which is seen 20 and 24 h after FCS stimulation, was prevented in presence of MMP9/13 (Figure 5C).

### MMP13 mediates cell proliferation in melanocytes and melanoma cells

Ilomastat efficiently inactivates MMP1, MMP2, MMP3, MMP8, and MMP9, while the only described targets of the MMP9/13 inhibitor are MMP9 and MMP13. Therefore we concluded that the effect of the MMP9/13 inhibitor is MMP13-specific. Supportingly, the application of another inhibitor (CP 471474), targeting MMP1, -2, 3, 9, and -13, as well as an independent MMP13-specific inhibitor (CL 82198) showed the same effect on the Hm and A375 cells (Additional file 5, Figure S4).

**Figure 6:**
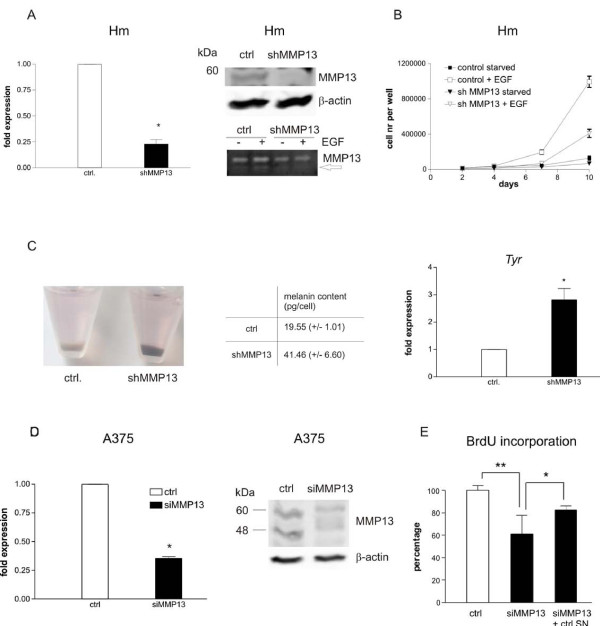
***MMP13 *knockdown in murine melanocytes and the human melanoma cell line A375 reduces proliferation**. A, left: Realtime-PCR of *Mmp13 *expression in melan-a Hm cells transgenic for a control pRetroSuper vector (ctrl.) compared to melan-a Hm cells expressing pRS-sh*Mmp13*. A, right, top: Western blot displaying the decrease of the pro-form of MMP13 after knockdown in murine melan-a Hm cells. Note that only the human, but not the murine active form is recognized by the antibody (also see D). A, right, bottom: Zymography of unconcentrated supernatant, showing a decreased MMP13 band after knockdown. B: Starved melan a Hm pRS and Hm pRS-sh*Mmp13 *cells were stimulated with EGF, and cell proliferation was measured after 2, 4, 6, 8 and 10 days. C, left: Appearance of cell pellets of EGF-stimulated Hm cells either transfected with the control vector or the pRS-sh*Mmp13 *vector. C, middle panel: Quantification of melanin in EGF-stimulated Hm cells either transfected with the control vector or the pRS-sh*Mmp13 *vector. C, right: Realtime PCR analysis of tyrosinase transcript levels in control Hm cells and Hm cells expressing pRS-sh*Mmp13*. β-actin was used as reference gene. D, left: Realtime-PCR of *MMP13 *expression in A375 cells transfected with siRNA against *MMP13 *or an siRNA control, 72 h after transfection. D, right: Western blot of MMP13, showing the full-length (60 kDa) and the active (48 kDa) form of the protein in A375 cells treated with control or *MMP13*-specific siRNA. E: BrdU incorporation of A375 cells transfected with control siRNA or *MMP13*-specific siRNA. The right bar shows the BrdU incorporation of A375 cells, treated with *MMP13*-specific siRNA, but in presence of the 48 h-conditioned supernatant from control siRNA-transfected cells. BrdU incorporation of the control cells is used as reference and is set as 100%. The assay was performed three times independently. *: p < 0.05 (Student's t test, paired, two-tailed)

To validate this, we transfected melan-a Hm cells with a retroviral plasmid expressing *Mmp13*-specific shRNA, which resulted in a reduction of *Mmp13 *expression on RNA and protein level (Figure 6A, left and middle panel). Melan-a Hm sh*MMP13 *cells proliferated much slower than cells expressing a control plasmid (Figure 6B). Interestingly, we also observed that *Mmp13 *downregulation went along with a strong increase in pigmentation, as visible by a 100% increase in melanin content (Figure 6C, left and middle panel). This was accompanied by enhanced levels of tyrosinase RNA (Figure 6C, right).

A similar approach was done with the human melanoma cell line A375. As several tested shRNA constructs did not efficiently knock down the gene, we used commercial siRNA for this cell line, which reduced *MMP13 *transcript levels to approx. 33% (Figure 6D, left). Western blot analysis also confirmed a reduction in the pro-and active forms of the protein, with 60 and 48 kDa, respectively (Figure 6D, right). Instead of the previously conducted long-term proliferation assays, we performed a BrdU incorporation assay as a measure of DNA replication 72 h after transfection of the respective siRNA (Figure 6E). Knockdown of *MMP13 *decreased BrdU incorporation to 60%. We also observed an increased fraction of si*MMP13*-transfected cells in the G0/G1 phase of the cell cycle when compared to control cells (Additional file 6, Figure S5). However, the effect was weaker than the effect seen in presence of the MMP 9/13 inhibitor displayed in figures 3C and 5C. Possibly, this is due to the incomplete *MMP13 *knockdown. It is also likely that the arrest is more enhanced in starved cells that are confronted with growth stimulus and MMP inhibitor at the same time. If *MMP13 *is knocked down in the normal growing cell culture, it may block cell cycle progression in general, irrespective of the cell cycle phase. This kind of behaviour is reminiscent of the effect of growth factor withdrawal, which can block the cell cycle in G1 and G2, and might point to the possibility that MMP13 releases an unidentified growth factor. To investigate whether the potential MMP13-dependent growth-promoting factor is secreted, we treated si*MMP13*-transfected A375 cells with conditioned supernatant from control siRNA-transfected cells. This could significantly restore BrdU incorporation to 80% of the control (Figure 6E), indicating the presence of a soluble growth-promoting factor.

In summary, these data indicate that MMP13 plays an important role in the growth factor-induced proliferation of melanocytes and melanoma cells as well as in the dedifferentiation of melanocytes.

## Discussion

In most melanomas, MMPs are aberrantly expressed. All MMPs upregulated in Hm cells were previously reported to be produced in melanoma, in particular MMP1 and -9 [29]. The cause of MMP expression in melanoma is largely unknown, but continuous ERK signalling, e.g. by autocrine FGF- or B-Raf^V600E^- signalling is responsible for their expression in some melanoma cell lines [30,31].

The generally favoured function of MMPs in melanoma progression is the remodelling of the extracellular matrix that enables both the transition of radial to vertical growth phase and angiogenesis in more advanced stages of the disease [30,32,33]. However, although tumor cells commonly express ample amounts of MMPs, MMP-independent migration was reported for melanoma, fibrosarcoma and breast cancer cells [25,26,34-36].

Consistent with the concept of MMP-independent migration, our data show that the EGF-induced upregulation of MMP13 in melanocytes supports cell cycle progression instead of invasive migration.

MMP13, also called collagenase-3, is expressed in a very restricted manner in the human body, but is often upregulated under pathological conditions, such as cancer and arthritis [37]. Under physiological conditions, it is mainly expressed in bone and cartilage, where it helps to remodel the growing tissue. Consequently, *MMP13-/- *mice show defects in growth plate cartilage and disturbed ossification, which is at least partly the result from interstitial collagen accumulation [38]. Hence, collagens, such as collagen II and IV, are the best investigated MMP13 targets [39,40]. However, the role of MMP13 in mediating melanocyte and melanoma cell proliferation as described in this manuscript is in line with emerging non-classical MMP functions in outside-in signalling and cell cycle control. The subsequent signal transduction events responsible for this process are unclear so far, but matrix- or cell surface proteins, either activated or made accessible by MMP13 dependent cleavage, may be involved. Generally, MMPs can release growth factors such as HB-EGF and TGF-α, but also secreted factors or proteins that can regulate growth factor availability, such as IGFBP1-, -3 and -5 and FGF receptor [41-47]. In squamous cell carcinoma, MMPs generate autocrine loops that are able to stimulate several receptors of the EGFR family [48]. It is well possible that a similar effect occurs MMP13-dependently in Hm and A375 cells. For MMP13, a wide substrate specificity was described, and several non-matrix substrates are already known, such as TGF-β, CTGF, fibrinogen and ICAM-1 [37,40,49-51]. Furthermore, MMP9 is a confirmed target of MMP13, and it is also involved in the cleavage of numerous substrates, including integrin precursors and LIF [52,53]. Whether these or yet unknown targets are responsible for proliferation in melanoma will be investigated in the future.

Interestingly, protein expression of MMP13 is absent from nevi, but was noted in almost 50% of cutaneous melanoma [54]. A functional role for stromal MMP13 in melanoma development was recently described in a *MMP13*-/- mouse model [55]. In these mice, B16F1 melanoma grafts displayed reduced tumor growth and strongly decreased metastasis and angiogenesis compared to wildtype mice. Together with our data, it appears that tumor cell - or stroma-derived MMP13 plays a role in several processes of melanoma development. This makes it a potentially attractive drug target. Selective MMP13-specific protease inhibitors are already developed [56,57] and are currently used in mouse models for arthritis [58]. In future studies, we will investigate the effect of specific MMP13 inhibitors in animal melanoma models.

## Conclusions

Our data demonstrate that MMP13 links growth-stimulatory signals such as EGF and FCS to cell cycle progression in melanocytes and melanoma cells and to dedifferentiation in melanocytes. The data indicate that the protease is important for migration-independent processes of melanoma formation, possibly by releasing a yet unidentified growth factor. As MMP13 also plays a role for melanoma progression and specific inhibitors are already developed, it might be considered as a target for the treatment of MMP13-sensitive melanoma.

## Methods

### Cell Culture

A375 cells were maintained in DMEM, 10% FCS in the presence of penicillin/streptomycin. Mouse melanocytes transgenic for the chimeric receptor HERmrk (melan-a Hm) or human EGFR (melan-a HER) were cultivated in DMEM, 10% FCS in the presence of cholera toxin (12 nM; Calbiochem), TPA (200 nM; Calbiochem) and penicillin/streptomycin [15]. Melan-a cells are a non-transformed cell line that are dependent on TPA for cell growth and proliferation. The following inhibitors were used and applied in the mentioned concentrations, unless stated otherwise:: AG1478 (20 μM); EGFR inhibitor, (10 μM), U0126 (10 μM); LY294002 (10 μM); PP2 (20 μM), MMPI 9/13 (10 μM), GM6001 - also called Ilomastat - (10 μM) (all from Calbiochem, Merck), PD184352 (2 μM) (Axon Medchem), CL 82198 (75 μM), CP 471474 (100 μM) (both from Tocris Bioscience). Ilomastat is an efficient inhibitor of MMP1, -2, -3, -8 and -9, CP 471474 inhibits MMP1, MMP2, MMP3, MMP9, and MMP13, MMPI 9/13 blocks MMP9 and MMP13 and CL 82198 specifically targets MMP13. Starving of cells was performed in DMEM medium containing no additives but 1.5% dialyzed FCS (Invitrogen), unless indicated otherwise. EGF (Tebu-Bio) was used in the concentrations indicated in the text and figure legends.

### Knockdown methods

For cloning of the sh*MMP13 *knockdown vector, the oligonucleotides gatccccGAGCACTACTTGAAATCATttcaagagaATGATTTCAAGTAGTGCTCtttttggaaa and agcttttccaaaaaGAGCACTACTTGAAATCATtctcttgaaATGATTTCAAGTAGTGCTCggg, containing the target sequence of murine *Mmp13 *[ENSMUSG00000050578], were annealed and cloned into pRetroSuper previously digested with BglII and HindIII. The resulting plasmid was retrovirally delivered into melan-a Hm cells and selected by puromycin treatment to obtain stable cellular expression. For human melanoma cells, commercially available control siRNA and siRNA against human *MMP13 *(Smart Pool On Target Plus, Thermo Scientific, [ENSG00000137745]) were used. siRNA was transfected using X-treme gene transfection reagent (Roche), according to the manufacturer's recommendations. Downregulation was monitored by realtime-PCR.

### Cell proliferation assay

Cells were starved for three days in DMEM containing 1.5% dialyzed FCS (Invitrogen) and seeded at 3 × 10^4 ^cells per well of a 6-well plate. Hm cells were treated with 100 ng/ml EGF, and A375 cells were treated with 10% FCS in absence or presence of 10 μM Ilomastat, 10 μM MMP9/13 inhibitor 1, or both. The controls were treated with the corresponding amount of DMSO. Cells were harvested by trypsinization after 2, 4, 6, 8, and 10 days, pelleted, resolved in PBS and counted under the microscope.

### BrdU incorporation assay

72 h after siRNA treatment, cells were incubated with 10 μM BrdU for 24 h. The following day, BrdU incorporation was quantified using a colorimetric BrdU cell proliferation ELISA, as recommended by the manufacturer (Roche).

### RNA isolation, reverse transcription and realtime PCR analysis

RNA isolation was performed using TrIR solution (ABGene) according to the manufacturer's instructions. 0.5-2 μg of whole RNA was reversely transcribed using the RevertAidTM First Strand cDNA Synthesis Kit (Fermentas). For the reverse transcription-PCR analyses of *Mmp1a/-b *[ENSMUSG00000043089], [ENSMUSG00000041620] expression in Hm cells, PCR was stopped after 30 PCR cycles and visualized on an agarose gel. β-actin was shown as control. For realtime PCR analysis, fluorescence-based quantitative realtime PCR was performed using the iCycler (Bio-Rad) for quantification of the following transcripts: murine *Mmp3 *[ENSMUSG00000043613], *Mmp9 *[ENSMUSG00000017737], *Mmp13 *(see above), *Tyr *[ENSMUSG00000004651], all additional genes from table 1, and well as human *MMP13 *(see above). β-*actin *[ENSMUSG00000029580] and ribosomal gene *S14 *[ENSG00000164587] were used as reference genes for murine and human genes, respectively. Relative expression levels were calculated applying REST software [59]. For all genes indicated, realtime analysis was performed at least three times independently from three different cDNA templates. The respective oligonucleotide sequences are available on request.

### Cell lysis and Western blot analysis

Cells were lysed in lysis buffer (20 mM HEPES (pH 7.8), 500 mM NaCl, 5 mM MgCl_2_, 5 mM KCl, 0.1% deoxycholate, 0.5% Nonidet-P40, 10 μg/ml aprotinin, 10 μg/ml leupeptin, 200 μM Na_3_VO_4_, 1 mM PMSF and 100 mM NaF). 50 μg of protein was resolved by SDS/PAGE and transferred to nitrocellulose according to standard Western blotting protocols. Anti-β-actin (C-4) and anti-ERK2 (C-14) antibodies were purchased from Santa Cruz Biotechnology. Anti-P-ERK1/2 (Thr202/Tyr204), anti-P-AKT (Ser473) and anti-cleaved caspase 3 (Asp175) antibodies were purchased from Cell Signaling/NEB, and anti-MMP-13 antibody was purchased from Abnova.

### Melanin quantification

Melan-a Hm cells from EGF-treated cell culture were trypsinized, and 5 × 10^5 ^cells were spun down in an Eppendorf centrifuge. The supernatant was discarded and the pellet was dissolved in 1 N NaOH. Melanin concentration was determined by measurement of optical density at 475 nm and compared to a standard curve obtained using synthetic melanin (Sigma). Pigment determination was performed three times independently.

### Zymographic analysis

FCS-free culture media of melan-a Hm cells, untreated or pretreated with EGF for two days, were harvested, adjusted according to the cell number and concentrated using Amicon Ultracel 10 k columns unless indicated otherwise. Samples were mixed with 2× loading buffer (50 mM Tris-HCl [pH 6.8], 10% [vol/vol] glycerol, 1% [wt/vol] SDS, 0.01% [wt/vol] bromophenol blue,) and resolved on an SDS-polyacrylamide gel containing 0.12 mg/ml gelatin (porcine skin type A; Sigma). Gels were soaked for 1 h in 2.5% Triton X-100, then washed twice with collagenase buffer (50 mM Tris-HCl [pH 7.6], 0.2 M NaCl, 5 mM CaCl_2_, 0.2% Brij 35), and incubated at 37°C for 18 h. Gels were then washed with distilled water and incubated in Coomassie brilliant blue staining solution (40% methanol, 10% acetic acid/0.025% Coomassie brilliant blue R-250) at room temperature for 2 h. Subsequently, gels were washed for 24 h in distilled water and scanned.

### Flow cytometry

Cells were starved for three days in 1.5% starving medium before being stimulated with 100 ng/ml EGF (Tebu-Bio) (Hm cells) or 10% FCS (A375). Cells were harvested after 0, 16, 20 and 24 h of stimulation and fixed in 70% ethanol. For flow cytometry analysis, DNA was stained with 69 mM propidium iodide in 38 mM sodium citrate and 100 mg/ml RNase A for 30 min at 37°C. Samples were analyzed in a Beckman Coulter Cytomics FC 500.

### Transwell migration assay

2,5 × 10^4 ^Hm cells were serum-starved in DMEM, 1% dialyzed FCS for 24 h and applied to the upper chamber of a transwell inlay (polycarbonate, 10 mm diameter, 8 μm pores, Nunc) in DMEM with 1% dialyzed FCS. Where indicated, transwell inlays were pre-coated with 3 μg/ml vitronectin (Biosource), 10 μg/ml collagen I (Upstate Biotechnology) or 10 μg/ml fibronectin (BD Biosciences), yielding fibrillar layers. The indicated concentrations of EGF were applied to the lower chamber, and inhibitors were applied in the given concentration to the upper and lower chamber. After 12 h, the transwell assay was stopped. The cells on the upper side of the membrane were removed with a cell scraper, before the membrane was fixed for 5 minutes in methanol and stained for 20 minutes with 2% crystal violet dissolved in 2% ethanol. The membranes were then washed with PBS and the number of cells on the lower side of the membrane was counted. The migration rate was determined in absolute numbers. At all conditions, the assay was performed at least three times independently.

### Collagen matrix migration assay and cell tracking

Cells were embedded within a 3D fibrillar collagen matrix (final collagen concentration: 1.67 mg/ml) and either overlaid with starving medium (DMEM containing 2.5% dialyzed serum (Invitrogen)) or starving medium containing 500 nM EGF, which was the optimal concentration for migration of Hm cells under these conditions. For the inhibition experiments, MEK inhibitor U0126, MMP inhibitors Ilomastat and MMP9/13 inhibitor I, alone or in combination, AG1478 or the respective amount of DMSO were added to the matrix and the starving medium. The collagen matrix component in the chamber was approximately 2/3 of the total volume, the medium supernatant was 1/3. The chamber was hermetically sealed with paraffine, incubated at 37°C for 48 h and migration was monitored by time-lapse videomicroscopy. Locomotor parameters were obtained by computer-assisted cell tracking and reconstruction of the xy coordinates of cell paths for a step interval of 4 minutes. For each condition, three independent samples were measured, and the speed was calculated for 40 randomly chosen cells per sample. The viability of the cells was >95% and did not change in presence of EGF or inhibitors.

## List of Abbreviations used

bFGF: basic fibroblast growth factor; BrdU: bromodeoxyuridine; Col I: collagen I; DMEM: Dulbecco's modified Eagle's medium; DMSO: dimethyl sulfoxide; EGF: epidermal growth factor; EGFR: epidermal growth factor receptor; FCS: fetal calf serum; Fn: fibronectin; HB-EGF: heparin-binding epidermal growth factor; HERmrk (or Hm): human EGF receptor-Xmrk chimeric protein; IGFBP: insulin-like growth factor binding protein; MAPK: mitogen-activated protein kinase; MEK: mitogen-activated protein kinase kinase; MMP: matrix metalloprotease; PBS: phosphate-buffered saline; PDGF: platelet-derived growth factor; PI3K: phosphoinositide-3-kinase; RTK: receptor tyrosine kinase; TGF-α (or -β): tumor growth factor α (or -β)); TPA: 12-O-tetradecanoyl-phorbol-13-acetate; Tyr: tyrosinase; Vn: vitronectin; WCL: whole cell lysate; Xmrk: *Xiphophorus *melanoma receptor kinase.

## Competing interests

The authors declare that they have no competing interests.

## Authors' contributions

SM and MS conceived and designed the experiments. SM performed and analyzed most experiments, with contributions from KW, MAK, EH, and AH. SM wrote the manuscript. MAK, SG, KW, PF and MS helped writing the manuscript and contributed important discussions. All authors read and approved the final manuscript.

## Supplementary Material

Additional file 1 **Figure S1 - Inhibition of MEK and PI3K in EGF-treated melan-a Hm cells**. A: Starved Hm cells were pretreated for 1 h with the indicated concentrations of U0126 or LY294002 before 100 ng/ml EGF was added for 10 minutes. Cells were lysed, and 50 μg was then subjected to Western blot analysis and probed with the indicated antibodies. ERK2 was used as control. B: Transwell migration assay of Hm cells seeded onto collagen I-coated inlays and stimulated with 1 ng/ml EGF, applied to the lower chamber. Cells were additionally treated with 10 μM LY294002 (LY), 2 μM PD184352 (PD) or a combination of LY294002 and PD184352 (LY + PD) (D). **: p < 0.001 (Student's t test, paired, two-tailed). Click here for file

Additional file 2 **Figure S2 - MAPK-dependent MMP expression in melan-a Hm and melan-a HER cells**. A: Starved Hm cells were pretreated for 1 h with 20 μM AG1478, 10 μM EGFR-I (both inhibitors targeting EGFR and HERmrk), 10 μM U0126 or 2 μM PD184352 (both MEK inhibitors) before 100 ng/ml EGF was added for 10 minutes. Cells were lysed, and 50 μg was then subjected to Western blot analysis and probed with the indicated antibodies. ERK2 was used as control. B: Hm cells were serum-starved for 24 h and subsequently left untreated or treated with 100 ng/ml EGF in presence or absence of 10 μM EGFR-I or 2 μM PD184352. After 8 h, cells were harvested and reverse transcription-PCR was performed for the indicated genes (35 cycles). C: as in A, but performed with melan-a HER cells. D: as in B, but performed with melan-a HER cells, and with additional treatment with the inhibitors AG1478 (20 μM) and U0126 (10 μM). E: The starved parental cell line melan-a was treated with EGF, and a reverse transcription PCR was performed for the indicated genes (35 cycles). Pc = positive control (EGF-treated Hm cells); nc = negative control (H_2_O). Click here for file

Additional file 3 **Movie S1 - Time-lapse movie of EGF-treated melan-a Hm cells**. Cells were visualized with an inverted microscope at 200-fold magnification and were photographed every 4 min for a total of 48 h. Click here for file

Additional file 4 **Figure S3 - Inhibition of MMP9 and MMP13 does not affect apoptosis in Hm and A375 cells**. A: Starved Hm cells were left untreated or were treated with 100 ng/ml EGF in the absence or presence of the MMP9/13 inhibitor for 24 and 48 h. Cells were then lysed and 50 μg of cell lysate was analyzed by Western blot. Apoptosis induction was investigated using an antibody against cleaved caspase 3. As apoptosis control, UV-irradiated A375 cells were used. ERK2 was used as loading control. B: as in A, but performed with A375 cells and 10% FCS as growth stimulus. Click here for file

Additional file 5 **Figure S4 - Inhibition of cell cycle progression by two additional MMP inhibitors**. A: Starved Hm cells were treated with EGF in the absence or presence of the general MMP inhibitor CP 471474 (100 μM) or the MMP13-selective inhibitor CL 82198 (75 μM) and incubated for 24 h. Their cell cycle state was analyzed by flow cytometry. B: as in A, but performed with A375 cells and FCS as growth stimulus. Click here for file

Additional file 6 **Figure S5 - Knockdown of *MMP13 *delays cell cycle progression in A375 cells**. A375 cells were transfected with control- or *MMP13*-specific siRNA for 72 h, and the cell cycle state was monitored by flow cytometry. Starved A375 cells served as control. Click here for file
