# Supplementary figures and images for: MMP13 mediates cell cycle progression in melanocytes and melanoma cells: in vitro studies of migration and proliferation
Source: Mol Cancer. 2010 Jul 28;9:201. doi: 10.1186/1476-4598-9-201 (PMC2915980; doi:10.1186/1476-4598-9-201)

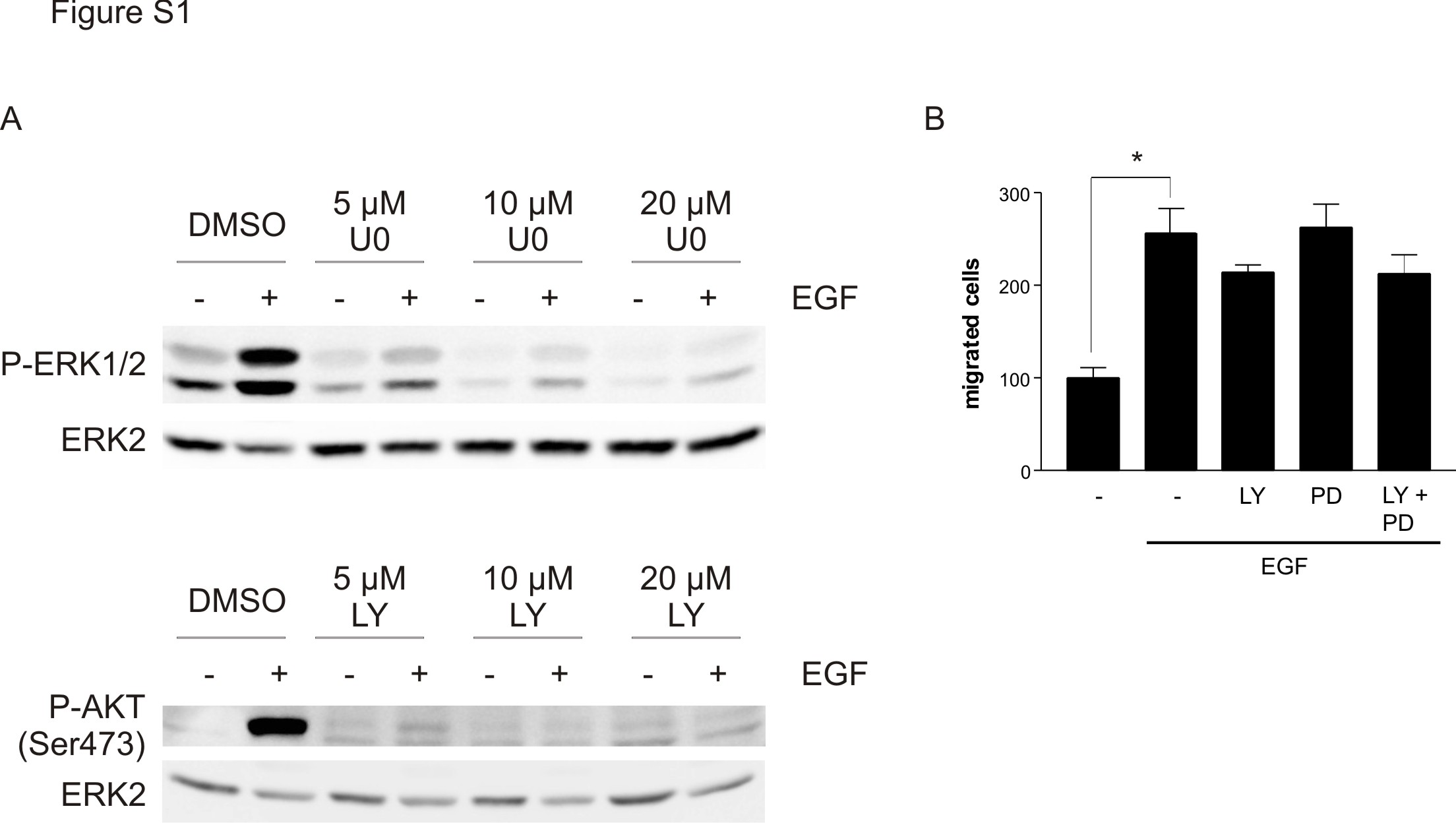

Supplement: Additional file 1 — Figure S1 - Inhibition of MEK and PI3K in EGF-treated melan-a Hm cells. A: Starved Hm cells were pretreated for 1 h with the indicated concentrations of U0126 or LY294002 before 100 ng/ml EGF was added for 10 minutes. Cells were lysed, and 50 μg was then subjected to Western blot analysis and probed with the indicated antibodies. ERK2 was used as control. B: Transwell migration assay of Hm cells seeded onto collagen I-coated inlays and stimulated with 1 ng/ml EGF, applied to the lower chamber. Cells were additionally treated with 10 μM LY294002 (LY), 2 μM PD184352 (PD) or a combination of LY294002 and PD184352 (LY + PD) (D). **: p < 0.001 (Student's t test, paired, two-tailed). [file 1476-4598-9-201-S1.JPEG]

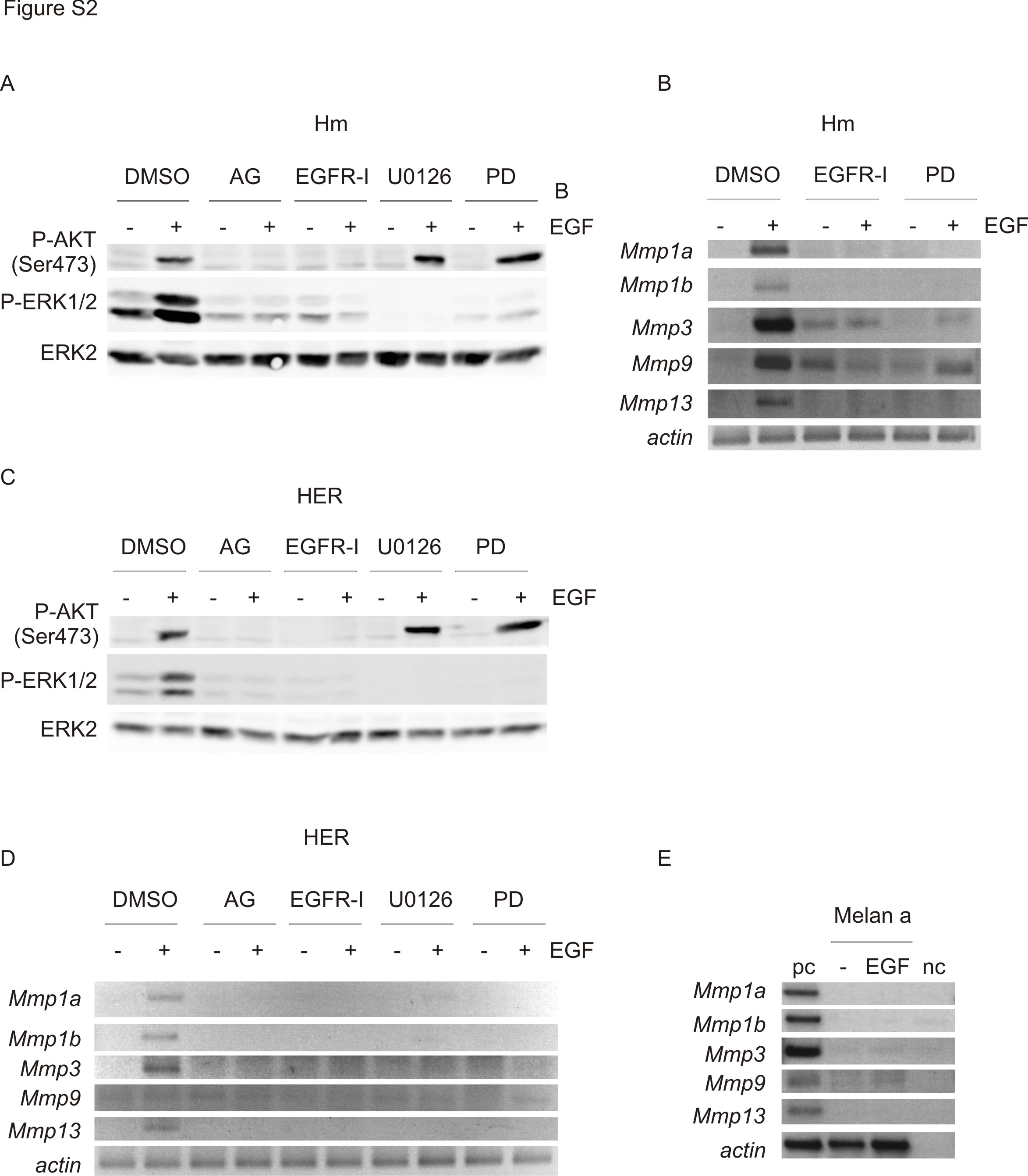

Supplement: Additional file 2 — Figure S2 - MAPK-dependent MMP expression in melan-a Hm and melan-a HER cells. A: Starved Hm cells were pretreated for 1 h with 20 μM AG1478, 10 μM EGFR-I (both inhibitors targeting EGFR and HERmrk), 10 μM U0126 or 2 μM PD184352 (both MEK inhibitors) before 100 ng/ml EGF was added for 10 minutes. Cells were lysed, and 50 μg was then subjected to Western blot analysis and probed with the indicated antibodies. ERK2 was used as control. B: Hm cells were serum-starved for 24 h and subsequently left untreated or treated with 100 ng/ml EGF in presence or absence of 10 μM EGFR-I or 2 μM PD184352. After 8 h, cells were harvested and reverse transcription-PCR was performed for the indicated genes (35 cycles). C: as in A, but performed with melan-a HER cells. D: as in B, but performed with melan-a HER cells, and with additional treatment with the inhibitors AG1478 (20 μM) and U0126 (10 μM). E: The starved parental cell line melan-a was treated with EGF, and a reverse transcription PCR was performed for the indicated genes (35 cycles). Pc = positive control (EGF-treated Hm cells); nc = negative control (H2O). [file 1476-4598-9-201-S2.JPEG]

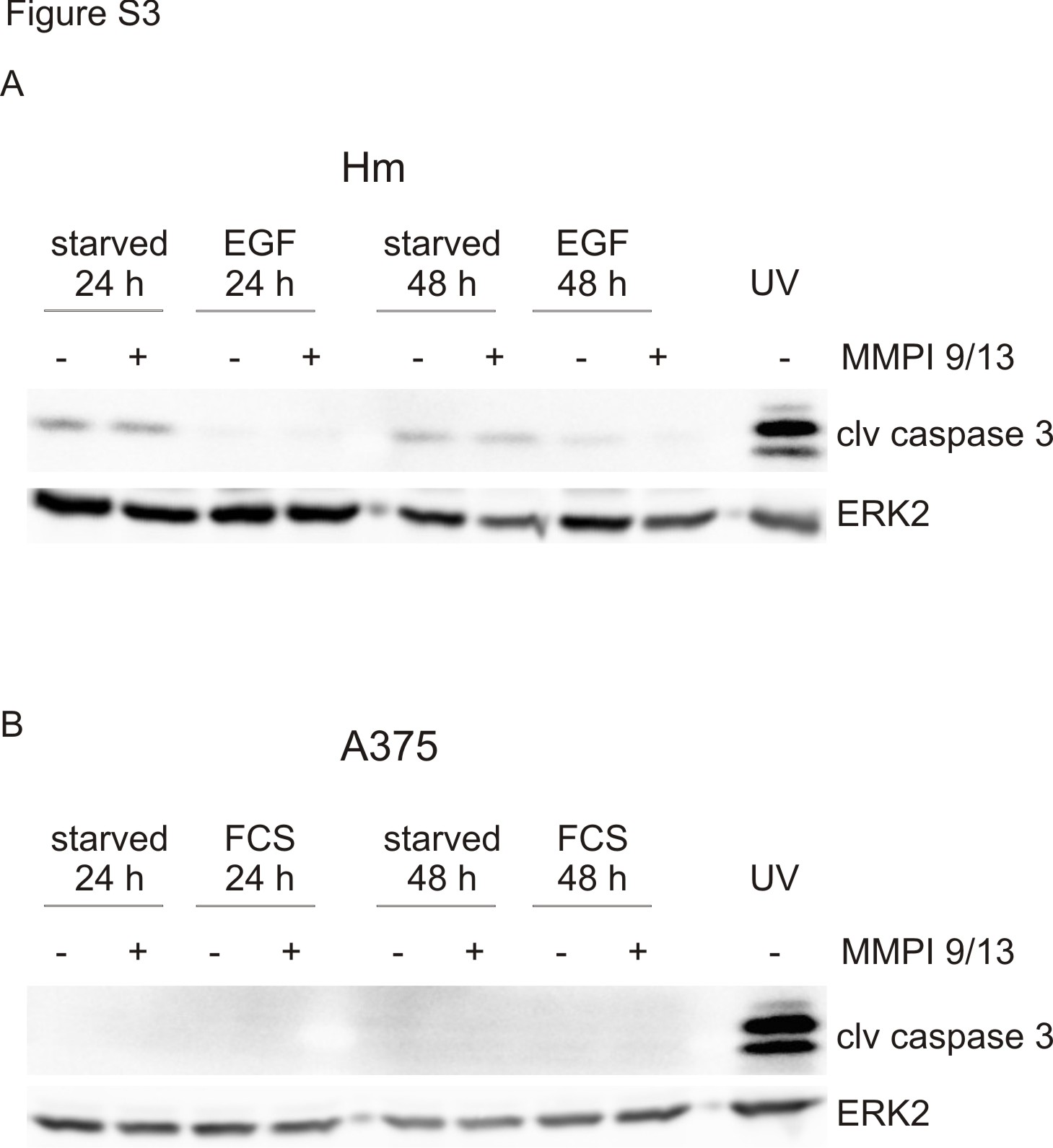

Supplement: Additional file 4 — Figure S3 - Inhibition of MMP9 and MMP13 does not affect apoptosis in Hm and A375 cells. A: Starved Hm cells were left untreated or were treated with 100 ng/ml EGF in the absence or presence of the MMP9/13 inhibitor for 24 and 48 h. Cells were then lysed and 50 μg of cell lysate was analyzed by Western blot. Apoptosis induction was investigated using an antibody against cleaved caspase 3. As apoptosis control, UV-irradiated A375 cells were used. ERK2 was used as loading control. B: as in A, but performed with A375 cells and 10% FCS as growth stimulus. [file 1476-4598-9-201-S4.JPEG]

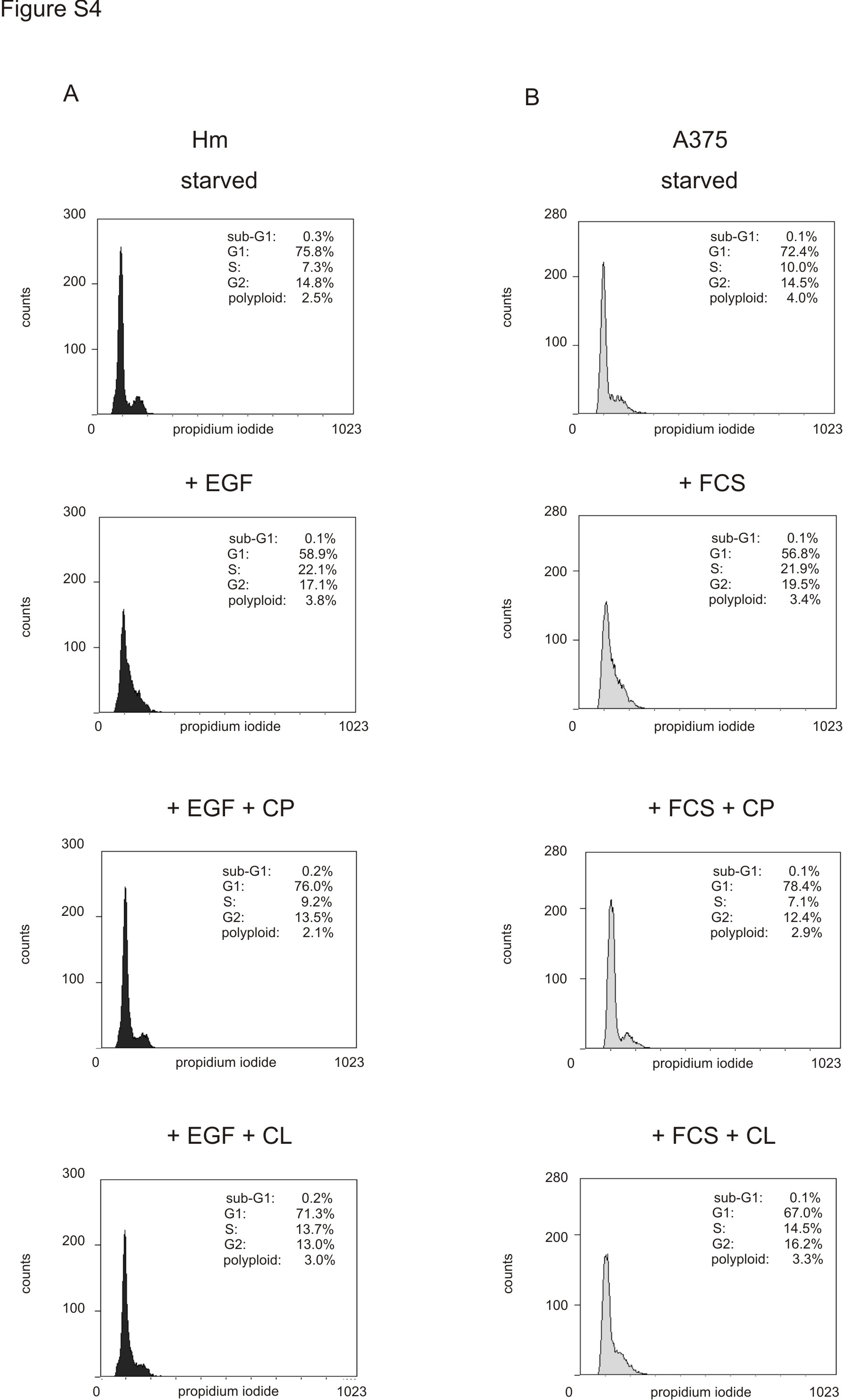

Supplement: Additional file 5 — Figure S4 - Inhibition of cell cycle progression by two additional MMP inhibitors. A: Starved Hm cells were treated with EGF in the absence or presence of the general MMP inhibitor CP 471474 (100 μM) or the MMP13-selective inhibitor CL 82198 (75 μM) and incubated for 24 h. Their cell cycle state was analyzed by flow cytometry. B: as in A, but performed with A375 cells and FCS as growth stimulus. [file 1476-4598-9-201-S5.JPEG]

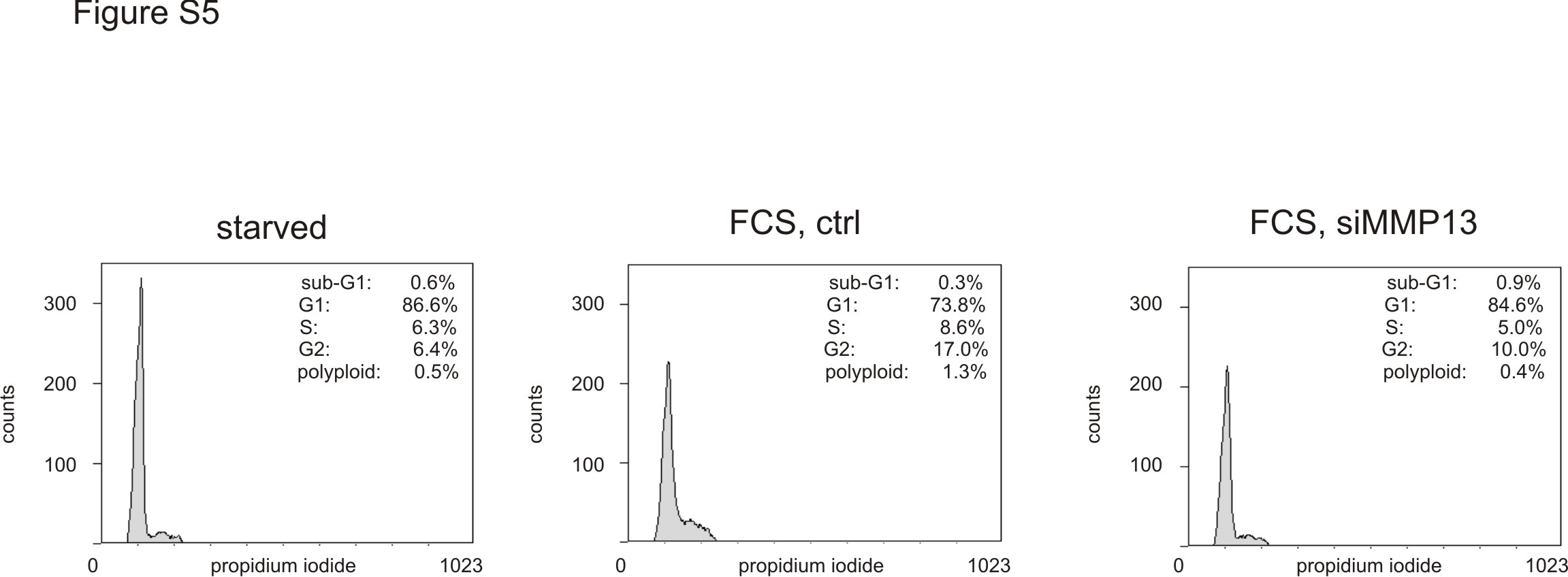

Supplement: Additional file 6 — Figure S5 - Knockdown of MMP13 delays cell cycle progression in A375 cells. A375 cells were transfected with control- or MMP13-specific siRNA for 72 h, and the cell cycle state was monitored by flow cytometry. Starved A375 cells served as control. [file 1476-4598-9-201-S6.JPEG]
